# Buckling-induced retraction of spherical shells: A study on the shape of aperture

**DOI:** 10.1038/srep11309

**Published:** 2015-06-22

**Authors:** Sen Lin, Yi Min Xie, Qing Li, Xiaodong Huang, Shiwei Zhou

**Affiliations:** 1 Centre for Innovative Structures and Materials, School of Civil, Environmental and Chemical Engineering, RMIT University, GPO Box 2476, Melbourne 3001, Australia; 2 School of Aerospace, Mechanical and Mechatronic Engineering, The University of Sydney, NSW 2006, Australia

## Abstract

Buckling of soft matter is ubiquitous in nature and has attracted increasing interest recently. This paper studies the retractile behaviors of a spherical shell perforated by sophisticated apertures, attributed to the buckling-induced large deformation. The buckling patterns observed in experiments were reproduced in computational modeling by imposing velocity-controlled loads and eigenmode-affine geometric imperfection. It was found that the buckling behaviors were topologically sensitive with respect to the shape of dimple (aperture). The shell with rounded-square apertures had the maximal volume retraction ratio as well as the lowest energy consumption. An effective experimental procedure was established and the simulation results were validated in this study.

Reconfigurable and reversible devices can rapidly respond to mechanical, thermal, chemical, electromagnetic and optical stimuli by changing their shapes and functionalities. Such active structures capable of smartly adapting to ambient environment widely exist in nature. For instance, some viruses can expand and retract their shell-like bodies by opening and closing the external apertures when the environmental pH changes^1^,^2^. Similar shape-invariant retraction of a ball-like structure can be achieved by devising a rigid network of struts connected with hinges, which is known as Hoberman Twist-O transforming sphere.

To avoid taking a complex configuration, researchers have recently developed a hinge-free spherical structure made of soft rubber with 24 evenly-distributed circular dimples on its external surface^3^. It was found that the thin ligaments separated by the dimples could be buckled and rotated when negative pressure was applied inside. Such a porous structure was termed as “buckliball”, which can gradually shrink into a smaller ball-like structure at about half of its original size. This morphological insensitivity to the external load is attributed to the dimples which are completely filled by the collapsed ligaments during the retraction. Full discussions of this amazing phenomena can be found from literature^3^. Subsequently to Shim *et al*.’s seminal work, the buckliball was extended to cylindrical coordinate system, in which a cylindrical shell is patterned with an array of voids. This new mechanism was referred as a “buckligami” because it can offer buckling-induced folding which was essentially akin to paper origami. Afterwards, Hecke *et al*. introduced the idea of controllable folding into the cubic lattice, forming nonperiodic structures with maximally auxetic yet isotropic properties^4^. It should be also noted that such buckling-induced failure can also be utilized to obtain extraordinarily strong load bearing capacity when flat sheets is folded in Ron Resch pattern^5^.

Buckliball is the first morphable structure that enables the buckling-relevant failures to be customized for the sake of specific functionalities. It spans a much wider spectrum of designing foldable structures such as drug delivery^6^, photonic crystals with tunable lensing effects^7^, soft robots^8^, and micro fabrication^9^. But these potential applications call for immediate actions to achieve dramatic and isotropic contraction because a larger ratio of volume retraction generally indicates a better performance (e.g. allowing the drug capsule passing through narrower vessel and robot squeezing into tinier space). There has been considerable effort towards improving such a ratio. For instance, as a type of buckliball applications, 3D structures made of soft materials and composed of elliptical pores possess a larger volume retraction ratio^10^. It was also reported that the structure consisted of the self-repeating buckliballs could exhibit extraordinary negative Poisson’s ratio if its volume retraction ratio becomes large enough^11^.

The volume retraction is resulted from buckling and post-buckling of the ligaments and thus its ratio depends on the thickness and size of the shell^12^,^13^,^14^, the number of dimples and the properties of constituent material. Unfortunately, the association of the buckling performance with the dimple geometry has not been completely studied yet. Considering the fact that structural optimization^15^,^16^, one of the most versatile and robust design methods mainly based on shape derivative and topological derivative^17^,^18^,^19^, has extensively illustrated the structural performance and material properties are highly affected by the shape and topology of their architectures^20^, it is pivotal to study the role of dimple shape.

In this study, various dimple (becomes an aperture if no membrane is glued inside) shapes defined by Gielis’ superformula were explored to seek for a larger volume retraction ratio. First, a computational model was created in a Finite Element Analysis (FEA) package (Abaqus) for buckling and post-buckling analysis by imposing a velocity field against the external shell surface. The performance of apertures in different shapes with the same surface area was investigated. Numerical tests demonstrate that both the linearly elastic (LE) and neo-Hookean (NH) material models are applicable for the constituent material of buckliball undergoing large but reversible deformation. Given that energy consumption exhibits a marked indicator to evaluate the efficiency of such a local folding mechanism, the densities of bending energy and stretching energy on the surface of buckliball were calculated in terms of LE model. It is finally identified that the buckliball with rounded-square apertures has the maximum volume retraction ratio. This spherical lattice structure was prototyped by using 3D printing technique and an experimental procedure was established to verify its buckling-induced retraction in this study.

## Methods

The buckling and post-buckling behaviors of a buckliball that is made of soft thin-walled spherical shell perforated with complex shapes, present significant challenges in analytical studies. Numerical simulation is therefore considered an effective alternative to investigation into a sophisticate relationship between the aperture shape and corresponding buckling-induced deformation.

### The geometry of buckliball

**Figure 1:**
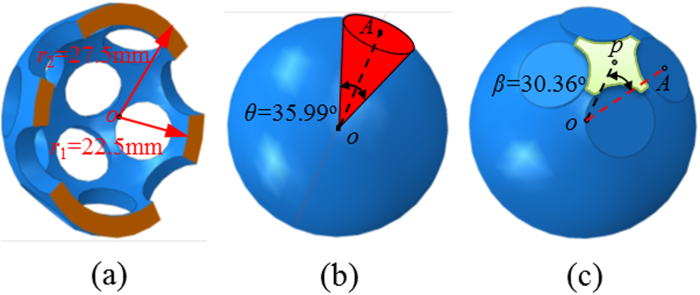
The modeling of a buckliball. (**a**) The schematic of a half buckliball; (**b**) The intersection between a cone and a spherical shell; (**c**) The distribution pattern of cones after being replicated four times.

**Figure 2:**
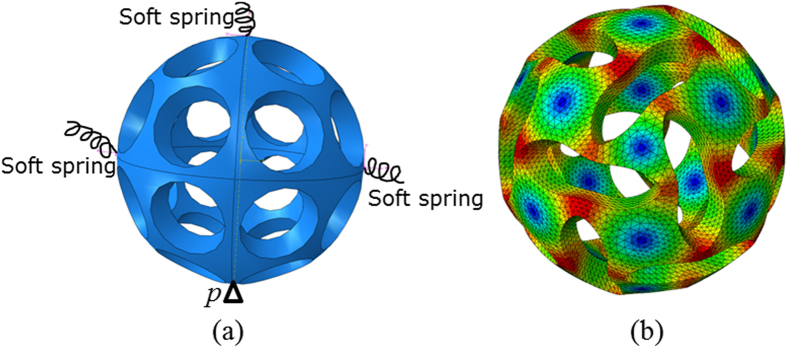
Linear buckling perturbation analysis (**a**) The boundary conditions; (**b**) Desired buckling pattern (the color represents the tangent deformation value).

In terms of the Jitterbug-like transformations^3^,^11^, only 6, 12, 24, 30 and 60 apertures are capable of producing desirable buckling patterns^21^. Following the previous studies, 24 apertures are considered in this study, which can be easily extended to the buckliballs featured with other number of apertures. The original architecture (Fig. 1a) consists of a spherical thin-walled shell perforated by identical solid cones with apexes at the center of a sphere (origin *O*). The inner and outer radii of the shell are *r*_1_ = 22.5 mm and *r*_2_ = 27.5 mm, respectively. By duplicating and reorienting the representative cone (red region in Fig. 1b) with respect to origin *O* three times, a pattern of cone distribution shown as in Fig. 1c was produced. The angle between the axis of each cone and the line connecting origin *O* to point *P* (the center of a cross-like solid part highlighted in light green color in Fig. 1c) is 30.36°. This pattern was duplicated six times to form 24 cones, which intersect with the shell to form the expected buckliball (Fig. 2a).

### The numerical simulation

The simulation of buckling and post-buckling process of the buckliball was conducted in Abaqus, a commercial finite element analysis tool widely used for nonlinear large deformation analysis. The abovementioned shell structure was adaptively discretized by 4-node linear tetrahedron elements (C3D4) and each node has 6 degrees of freedom. Such a mesh allows the buckling and post-buckling are simulated accurately and efficiently^22^. We modeled the isotropic rubber material as Neo Hookean elasticity with *C*_*10*_ = *G*/2 = 117,466 Pa and *D*_*1*_ = 2/*κ* = 3.45 × 10^−7^ Pa^−1^ (*G* and *κ* stand for shear modulus and bulk modulus, respectively), corresponding to Lamé constants *λ*_c_ = 5.64 MPa and *ν* = 0.48. The coefficients in such a hyperelastic model were measured in experiment in the supplementary information. Although the non-linearly elastic rubber is generally modeled by NH model, numerical simulation summarized in the supplement, together with previous investigation^23^, shows the use of LE model only slightly perturbs the buckling performance.

In contrast to previous work in which a layer of membrane is glued on the inner surface to produce airtight buckliball so that negative pressure can be easily obtained via pumping out the encapsulated air in experiment^3^, a membrane-free structure that is closer to the anticipated applications was studied herein. In the numerical simulation an improved volume control method was utilized to function as negative pressure. Such an open structure also steers away from the intricate contact problems between the membrane and shell. In this method a radial displacement field was applied to the external surface which serves as stress perturbation for characterizing buckling mode. To avoid rigid body motion, a pin support was specified at the bottom of buckliball (point *P* in Fig. 2a) while the leftmost, rightmost points and the vertex were supported by soft springs (Fig. 2a). Due to the prescription of extremely low stiffness (*K* = 1 N/m) of the springs, the influence of these additional constraints on the mechanical responses is negligible. Noted that the number of springs is considered as insufficient (less than 3) or redundant (more than 3) as improper constraints could result in either unstable or over-constrained structures, respectively. The nonlinearity of large deformation was solved by an implicit procedure in finite element analysis.

In addition to material imperfection, the commonly adopted approach in buckling analysis is geometric imperfection, whose form and amplitude have to be specified based on certain assumptions or prior knowledge^24^. Since the most rational imperfection^25^ statistically relies on a clunky collection of surveys on the prototyped buckliball, the initial geometric imperfections are often assumed to be one of the most influential eigenmode shapes^26^. It was found that if the imperfection vector aligns with the direction of the post-buckling path, the buckling load of the system would reach its global minimum^27^. Of these 20 eigenmode-affine shapes obtained from the calculation based on Lanczos algorithm^28^, it was found that only the one characterized with the second minimum eigenvalue *λ* = 4.644 × 10^−4^ is capable of producing the expected buckling pattern shown as in Fig. 2b (the red/blue colour represents large/small tangent deformation due to isotropic radial displacement imposed on the external surface).

**Figure 3:**
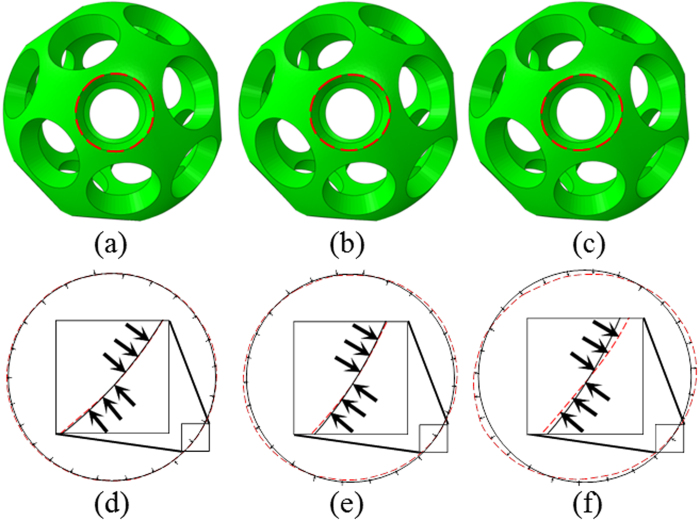
The eigenmode-affine shapes with *λ* = 4.644 × 10^−4^ and: (**a**) *α* = 0.01*t*; (**b**) *α* = 0.05*t*; (**c**) *α* = 0.1*t*; The zoomed-in aperture shapes with (**d**) *α* = 0.01*t*; (**e**) *α* = 0.05*t*; (**f**) *α* = 0.1*t*. (*t* denotes the thickness of shell)

The amplitude of geometric imperfection *α* has a significant effect on the buckling process as it substantially alters the shape of initial aperture and results in eccentricity^29^. In the previous studies^3^, Shim *et al*. concluded that the magnitude should be evaluated in accordance with the shell thickness. For the shell thickness *t* = 5 mm, the buckling of this spherical structure is robust and stable as long as *α* ≤ 0.1*t*. To conduct the numerical simulations of the buckling patterns of the buckliballs, weights of *α* = 0.01*t*, *α* = 0.05*t* and *α* = 0.1*t* were most often adopted to trigger the proper buckling^3^. To explore the effect of this amplitude, we also tested some smaller values such as *α* = 0.008*t* and *α* = 0.005*t*. However, we found an imperfection smaller than *α* = 0.01*t* could not trigger a desired buckling pattern. Consequently, *α* = 0.01*t* was considered to be the smallest value to ensure the occurrence of buckling. These buckliballs with geometrical imperfection are illustrated in Fig. 3a–c and one of their apertures encircled by the red dashed curve is correspondingly zoomed in Fig. 3d–f. The dark solid lines in Fig. 3d–f represent the boundaries without geometrical imperfection and the black arrows indicate the deformation tendency for the aperture edges.

In the improved volume control method, a velocity field is imposed on the external shell surface to replace the displacement-based stimulus. The relationship of velocity field *V* with time *τ* is given as:


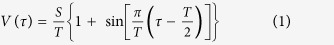


where *T* denotes the end time at which the apertures begin to self-contact. The integration of Eq. (1) from *τ* = 0 to *T* equals to the retraction distance of the external surface in the radial direction. Such a velocity definition guarantees nearly zero acceleration at the beginning and the end of the simulation^30^. To accommodate the velocity of deformation in simulation, the finite element model should have a minimum element size (*L*_*e*_) of 0.4 mm, the undamped elastic wave speed can be defined as *C*_*d*_ = (*E/ρ*)^1/2^, which is about 24.77 m/s. Noted the Young’s modulus and density are represented as *E* and density ρ, respectively. The estimated stability limit (*Δτ*_*stable*_ = *L*_*e*_/*C*_*d*_) is about 1.6 × 10^−5^ s. To ensure quasi-static conditions, the minimum time increment should not exceed the stability limit during the whole simulation^31^. As a result, such a velocity-controlled method enabled us to keep the kinetic energy negligible compared with the strain energy and therefore dramatic retraction caused by a large acceleration can be avoided.

**Figure 4:**
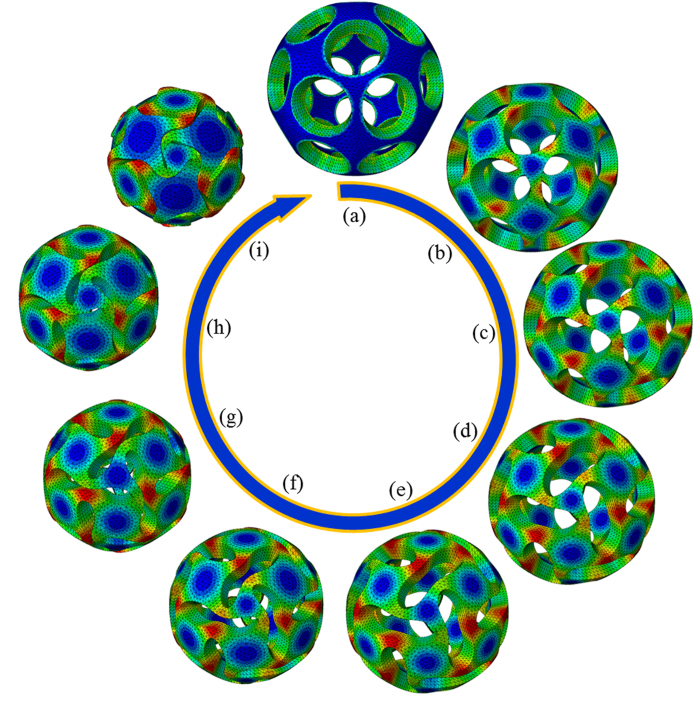
The buckling process of a typical buckliball with the geometric imperfection shown as in Fig. 3a. (the colors represent the stress)

The snapshots of the buckling process in Fig. 4 clearly show that the volumetric changes are entirely yielded by the distortion and rotation of the apertures. In the beginning, the structure exhibits a uniform shrinkage. All apertures remain circular shapes in this stage (Fig. 4a). Afterwards, the apertures deform to ellipses (Fig. 4b) and subsequently, they are transformed into peanut-like shapes, where their central part gradually becomes narrower and narrower until coming into contact (Fig. 4c–f). In these deformation stages, the apertures experience severe distortions as they rotate dramatically. Finally the apertures gradually close (Fig. 4g,h) and the structure turns into a nearly seamless sphere (Fig. 4i).

Without considering the thin layer of membrane, the void volume fraction (the volume ratio between the inner cavity and the external sphere with radius *r*_2_ = 27.5) is * ψ* = 0.59. Considering the fact that the buckliball approximately retains a spherical shape in the whole buckling process, the ratio of volume retraction *V*_r_ is derived as


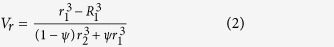


where *R*_1_ = 13.27 mm is the obtained inner radius of the buckled shell. According to Eq. (2), the maximal attainable volume retraction ratio should be *V*_max_ = 59.23%. It is interesting to note that in the previous work^3^ for a buckliball as shown in Fig 1a, *V*_r_ = 45.80%. From this perspective, more work is in demand to approach to the theoretical limit.

**Figure 5:**
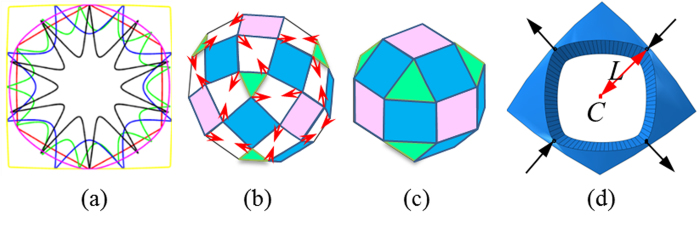
The investigation into aperture shape (**a**) A family of Gielis’ shapes; (**b**) Schematic of an expanded Jitterbug-like polyhedron; (**c**) A folded Jitterbug-like polyhedron; (**d**) The distance between the aperture center and one representative corner.

To produce larger retraction ratio, the base surface of the cones is not a circle but the shape determined by Gielis’ superformula^32^ in the geometrical modelling. In 2D, the radial function of Gielis’ shapes is given as:


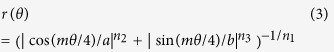


where −*π* ≤ *θ* ≤ *π* is used to locate the points on the shape by *x* = *r*(*θ*)cos(*θ*) and *y* = *r*(*θ*)sin(*θ*) in a cylindrical coordinate system. The number of rotational symmetries (vertices) of the Gielis’ shape is determined by parameter *m* and the size of shape is affected by *a* and *b*. These size factors were not in use herein as the apertures, regardless of shape, should have the same area as the original circle for the sake of comparisons of equality. To achieve such an equivalent matching, the size of shape was firstly estimated and then enlarged or reduced to achieve the same area as the original one. A variety of shapes can be produced by varying *m*, *n*_1_, *n*_2_ and *n*_3_ as shown in Fig. 5a. It is noted that in addition to common shapes such as the conventional square, circular and regular hexagon, complex geometries can also be generated. Because complex shapes would be difficult to manufacture, only polygon-like (e.g. square, hexagon and octagon) shapes were explored in this study.

## Results

### The study on volume retraction ratio

**Figure 6:**
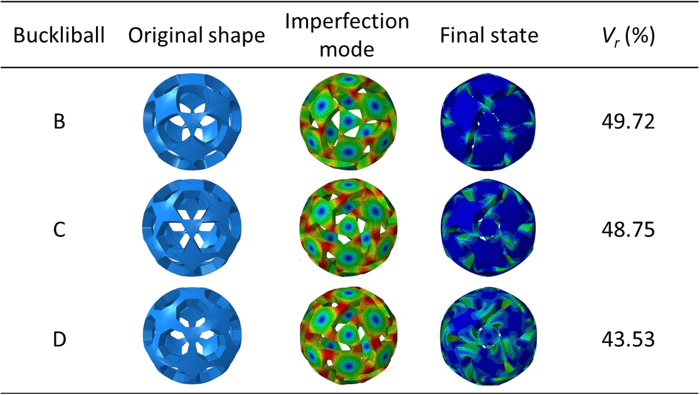
The simulation results of three basic shape apertures.

Figure 6 summarizes the aperture shape (2^nd^ column), imperfection mode (3^rd^ column) and final deformed shape (4^th^ column) for three types of buckliball perforated by different cones. In the 3^rd^ column, the green part indicates high stress area while blue for low stress area. The volume retraction ratio is given in the 5^th^ column. By superimposing eigenmode shape (3^rd^ column) weighted by *α* = 0.01*t* onto the initial structure, expected buckling mode can be produced accordingly.

Being a prior pattern for buckliball^33^, square apertures naturally form a Jitterbug-like polyhedron comprised of 20 squares and 8 triangles and constitute an interesting patterned shell (Fig. 5b). The movements of its nodes are schematically illustrated by the red arrows in Fig. 5b when this structure is compressed radially. As a result, the light green triangles and pink squares rotate counter-clockwisely while the blue squares rotate clockwisely. Theoretically, such a device can transform into a smaller polyhedron on which the initial white trapezoids eventually disappear^21^ (Fig. 5c). However, a buckliball derived from this model leads to singularity in numerical simulation due to the existence of single-point joints (the nodes in Fig. 5b). The rounded corners of square-like apertures produced by Gielis’ superformula (named Buckliball-B to distinguish from the original Buckliball-A with circular apertures in Fig. 4a) could avoid such unfavorable joints and this newly-developed buckliball (the structure in the first row and the second column in Fig. 6) largely preserves the same deformation patterns as the ideal Jitterbug-like polyhedron does. As a result, the volume retraction ratio is evidently improved from *V*_r_ = 45.80% to *V*_r_ = 49.72%, increasing by 8.56%. In the simulation, this model was discretized into 760,000 finite elements and it took about 12 hours on a workstation with 10-core 3.10 GHz processing units to solve. As in Fig. 6, the buckliballs with the apertures of the hexagon-like (Buckliball-C) and octagon-like shapes (Buckliball-D) were also investigated and the corresponding volume retraction ratios are *V*_r_ = 48.75% and *V*_r_ = 43.53%, respectively.

The difference in volume retraction ratios of these buckliballs is partially attributed to the distance *L* between the aperture center *C* and the point on the aperture edges at which the largest deformation takes place. From the 4^th^ column in Fig. 6, it is seen that the maximal deformation occurs at the vertices of Buckliball-B and Buckliball-C’s apertures. But for the aperture of Buckliball-D’s, the middle points of one pair of the opposite edges generate maximal deformation. The distance is *L* = 9.45 mm, *L* = 9.35 mm and *L* = 8.00 mm for Buckliball-B, C, and D, respectively. For Buckliball-A, the distance is equal to the radius of circle with 8.46 mm. According to the moving tendency (illustrated by the black arrows in Fig. 5d) of the points for the maximal deformation, larger *L* allows the apertures to be more inhomogeneously distorted and more completely closed. Therefore, the Buckliballs-B and C in Fig. 6 have distinctly larger volume retraction ratios than the Buckliball-A. The 3^rd^ row of Buckliball-D has the minimal volume retraction ratio simply because it has the shortest distance *L* = 8.00 mm. A sensible explanation in accordance with the abovementioned comparison is that the edges of apertures in some shapes are more likely to be self-contacted, prematurely terminating the retraction process before the apertures are completely closed.

### Energy analysis

Energy consumption in the deformation process was long considered a significant factor to measure the deformability of thin shells. Here energy distribution on the external surface was explored using Kirchhoff-Love’s theory based mechanics^34^, which bridges the buckling and post-buckling performance to volume retraction. If the shell thickness *t* is less than 10% of its radius, the energy consumed in the deformation process can be separated into stretching and bending energies, given as^35^:


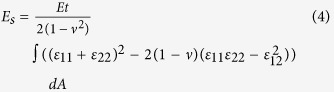



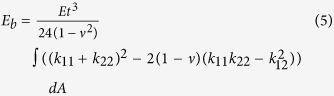


where *ε*_*ij*_ denotes the strain tensor and *k*_*ij*_ = *K*_*ij*_−*K*^0^_*ij*_ represents the change of the curvature tensor. It is noted that the initial curvature tensor *K*^0^_ij_ is related to the mean curvature *K*^0^_*ij*_ = *δ*_*ij*_*K*^0^ at a surface point where a local coordinate system is established in the tangent plane. *δ*_*ij*_ is the Kronecker’s delta. The symmetric curvature tensor *K*_*ij*_ has two normal curvatures termed as *k*_11_ and *k*_22_, and the two twist curvatures are identical as *k*_12_ = *k*_21_. To meet the assumptions of Kirchhoff-Love’s theory, the simulation was conducted by using LE-based material model, whose Young’s modulus *E* and Poisson’s *ν* are extracted from the coefficients in the NH model.

In order to calculate the stretching energy *E*_s_ and bending energy *E*_b_, a virtual shell, whose neutral surface is the external area of the buckliball was constructed. The thickness of the shell *t* = 0.45 mm is the average height of the elements on the most external layer of the shell. For this shell, the ratio of thickness to radius is less than 0.1 and therefore Eqs. (4, 5) hold effective and should be applicable.

**Figure 7:**
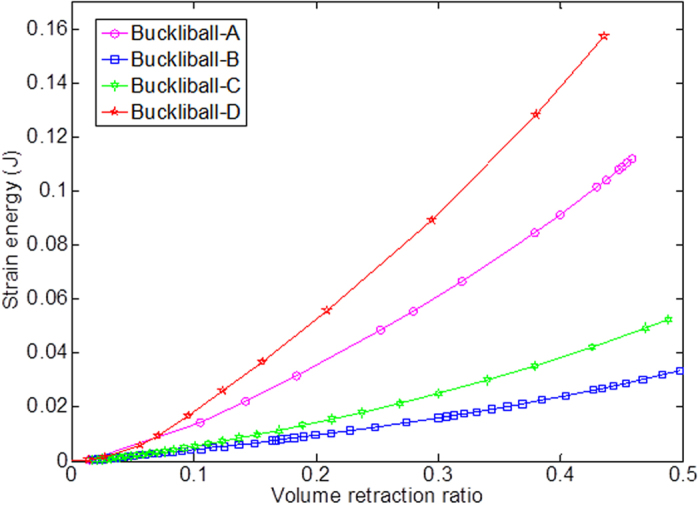
The energy consumption versus the volume retraction ratio for different buckliball configurations.

As shown in Fig. 7, Buckliball-B takes 0.0334 J energy in the whole deformation process, evidently smaller than the energy consumption of Buckliball-A (0.1119 J), Buckliball-C (0.0525 J) and Buckliball-D (0.1572 J). Such an unusual behavior of energy consumption is in conflict with the common sense that large deformation (in terms of high *V*_r_) usually consumes more energy. By observing the final appearance of the buckled structure for Buckliball-B and C, it can be seen that the stress is largely concentrated on the narrow joints, indicating that rest parts of the buckliball surface (e.g. the square and triangle patches on the surface of Buckliball-B) only move rigidly in radial direction and rotate in the tangent planes. In contrast, the corresponding regions of Buckliball-A and Buckliball-D suffer from substantially high stress and distortion (see the deformation in 3^rd^ row in Fig. 6). The energy consumed for deforming these parts makes no much contribution to the increase in volume retraction ratio and therefore is not considered effective.

**Figure 8:**
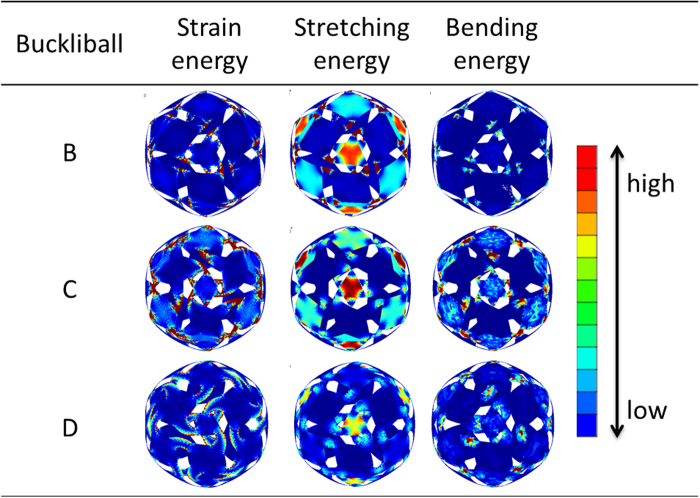
The magnitude of energy for three Buckliball configurations.

Figure 8 illustrates the strain energy, stretching energy and bending energy distributions on the external surface for the buckliballs given in Fig. 6. The strain energies of these three buckliballs mainly consist of the stretching energy, as the stretching energy is found to be 1,000 times higher than the bending energy. In these energy contours, the red parts represent high energy density, while the blue parts stand for low energy density. For the stretching energy distribution shown in the 3^rd^ column in Fig. 8, the energy mainly concentrates in the ligaments of Buckliball-B which undergo significant distortion, while the triangle and square patches consume a small amount of stretching energy. It means that the stretching energy has been efficiently utilized for triggering the buckling. For the Buckliball-C and D, the highest stretching energy also locates at the ligaments. However, it is only slightly higher than the energy in those square patches, which indicates that a large amount of energy is consumed for stretching those parts without much contribution to the desired buckling. The bending energy (4^th^ column in Fig. 8) is relatively low and uniform compared with the stretching energy, but it becomes high in the severely-deformed regions.

### Experimental validation

To experimentally validate the simulation results, the buckliball-B was fabricated using 3D printing technique. Because of the 64% elongation at break (the ratio of deformed length to initial length) and 0.44 MPa tensile strength, the rubber-like constituent material (FLX9840-DM) capable of withstanding large deformation in the buckling process was selected as the constituent material.

The Objet Connex350, a 3D multi-material printing system, was used to prototype the buckliball model as it allows a realistic model to be produced with acceptable accuracy in both geometric dimension and mechanical properties^36^. The process began with slicing the model into a series of closely spaced horizontal planes for the subsequent layer-by-layer printing process. To support the constituent material in printing semi-finished regions of the model which were temporally unstable, the supporting material (FullCure 705) was printed to fill the void space. It took about two hours to produce a cubic into which the buckliball was embedded. Later, the supporting material was melted into water and the sample was sculptured as anticipated. To trigger buckling more easily and obtain desirable buckling pattern, the size of prototyped sample was magnified by 1.8 times with the thickness *t* = 9 ± 0.1 mm and outer radius *r*_2_ = 49.5 ± 0.2 mm.

**Figure 9:**
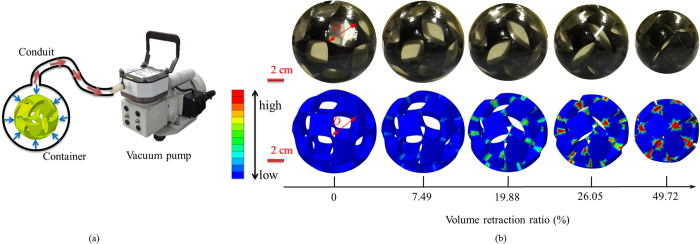
Experimental validation (**a**) A schematic of experimental validation; (**b**) The snapshots of experimental test and numerical simulation during buckling process. Photo courtesy of Sen Lin.

The most intuitive and direct method of radially compressing the specimen was to impose negative pressure against a membrane that is tightly glued on the inner surface. However, such a loading procedure resulted in the crumple of membrane which would hinder the retraction of buckliball. To tackle this problem, a new experimental scheme as shown in Fig. 9a was established. In order to observe the deformation clearly, the prototyped sample was placed into a thin latex rubber container whose inner surface tightly wrapped the sample in the beginning. When water was poured into the container which was connected to a vacuum pump through a conduit, the buckliball expanded and restored its original shape. When the pump sucked water from the container, uniform pressure was generated on the external surface of the buckliball due to the shrinkage of the container. Consequently, the expected buckling pattern was achieved. To avoid tilting of the buckliball, the whole system was placed on a frictionless horizontal plate. In addition, the conduit was perpendicular to the outlet of the container as shown in Fig. 9a. To be consistent with the velocity used in numerical simulation, the radial shrinkage of the container was attentively controlled by the pump at a constant speed of 12.4 mm/min, equivalent to decrease in the ratio of the outer radius of the shell to its original size at a rate of 0.209% per second. The whole deformation process was taken about 2 minutes and recorded from the front of shell. (The video is provided online) In order to account for experimental errors, the test was repeated three times by infilling and sucking the water.

It is interesting to make a comparison between physical test and numerical simulation in view of the fact that the elusive buckling behaviors are hardly to be predicted precisely. In Fig. 9b, the top snapshots were captured from the experiment at several successive timestamps, while the bottom images were obtained from simulation at the stages in which the buckliball retracted to similar volume retraction ratios to the experimental counterparts. This comparison illustrates that the numerical simulation largely agree with the physical test. The color contours plotted in the bottom of Fig. 9b represent the strain energy distribution, which shows that the stretching side of ligament undergoes higher strain energy than the bending side.

**Figure 10:**
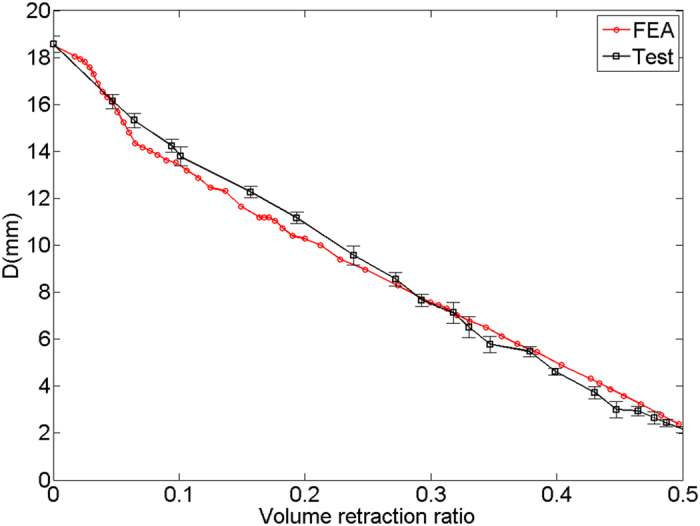
Comparison of the experimental and numerical results of the *D* value versus volume retraction ratio.

Additionally, to further quantify the difference between the simulation and the test, the deformed experimental configurations taken by camera were imported into AutoCAD and analyzed by digital image processing technique. With this method, the movements of the opposite corners approaching to each other during the test (marked as the red points in Fig. 9b) could be tracked. The schematic definition of the distance *D* = *2L* between the red points was estimated by measuring its projected length; while the diameter of the shell was estimated by measuring its projected area. Thereafter, the quantitative variations of *D* during the processes of experimental test and numerical simulation were compared in Fig. 10, in which the error bars represent the standard deviation of *D* in the experiment. It is seen that in terms of the value of *D*, two sets of data denoted by red and dark curves are quite close and the maximum difference is merely 5.88%. Fig. 10 also reveals the diameter *D* decreases almost linearly from 18.57 mm to 2.38 mm when the volume retraction ratio increases from 0 to nearly 50%.

## Discussion

In this work we have studied how to simulate the buckling-induced retraction for soft buckliballs featured with different apertures on their spherical shell. In order to avoid dramatic retraction rate and maintain the quasi-static conditions, a velocity-controlled loading approach characterized with nearly zero acceleration during the simulation was developed to replace previous pressure-controlled method. It was found that the most desirable buckling mode used as geometric imperfection was the eigenmode shape corresponding to the second eigenvalue. This imperfection aligns with the direction of the post-buckling path, which helps the buckling load reach its global minimum.

This study investigated what kind of aperture shape defined by Gielis’ superformula could generate a higher volume retraction ratio for the buckliball. We found the rounded square aperture allows this ratio approaches to 49.72%, which is 8.56% larger than that of buckliball perforated by circular apertures. Further studies are expected to seek the maximum volume retraction ratio and/or minimum energy consumption for the given materials and weight. This work concluded that the difference in volume retraction ratio highly relies on the distance between the aperture center and the points on their edges. Generally, a longer distance allows the apertures to be more inhomogeneously distorted and more completely closed in the end. Based on the analysis of energy consumption in the deformation process, we noted that the stress of buckliball offering maximal volume retraction ratio mainly concentrates in the joints, leading to radial rigid motion and tangential rotation for the patches connected by the joints. It was shown that the stretching energy dominates the buckling process as it is 1,000 times higher than the bending energy. In terms of the comparison of simulation results, it is believed that both the NH and LE material constitutive rules are applicable for modeling the reversible large deformation for the buckliball. The buckliball with the maximal volume retraction ratio was fabricated using 3D printing technique and we developed an effective experimental scheme to validate the numerical simulation.

Overall this study established a fundamental framework to simulate, design and validate the buckling and post-buckling behaviours for a soft shell-like structure. The parametric and shape controls allow to obtaining buckling mode on demand, thereby tailoring the structure for desirable deformation and energy absorption. The improved volume retraction ratio for the buckliball will benefit to the potential applications involving drug delivery systems, arterial stents, deployable structures and soft robots.

## Additional Information

**How to cite this article**: Lin, S. *et al*. Buckling-induced retraction of spherical shells: A study on the shape of aperture. *Sci. Rep*. **5**, 11309; doi: 10.1038/srep11309 (2015).

## Supplementary Material

Supplementary Information

Supplementary Video S1

## References

[b1] Suh W. H., Jang A. R., Suh Y. H. & Suslick K. S. Porous, hollow, and ball-in-ball metal oxide microspheres: Preparation, endocytosis, and cytotoxicity. Adv. Mater. 18, 1832–1837 (2006).

[b2] Lidmar J., Mirny L. & Nelson D. R. Virus shapes and buckling transitions in spherical shells. Phys. Rev. E 68, 051910 (2003). 10.1103/PhysRevE.68.051910 14682823

[b3] Shim J. . Buckling-induced encapsulation of structured elastic shells under pressure. Proc. Natl. Acad. Sci. U.S.A. 109, 5978–5983 (2012). 22451901 10.1073/pnas.1115674109 PMC3341048

[b4] van Hecke M., Coulais C. & Florijn B. 3D buckligami: combinatorial mechanical metamaterials. in the Society of Engineering Science 51st Annual Technical Meeting. (ed Zavattieri P., Bajaj A., Koslowski M. & Siegmund T. ) (Purdue University Libraries Scholarly Publishing Services, 2014).

[b5] Lv C. . Origami based Mechanical Metamaterials. Sci. Rep. 4, 5979 (2014). 25099402 10.1038/srep05979 PMC4124469

[b6] Zhu Y. . Stimuli-responsive controlled drug release from a hollow mesoporous silica sphere/polyelectrolyte multilayer core-shell structure. Angew. Chem. 44, 5083–5087 (2005). 16015668 10.1002/anie.200501500

[b7] Wu Q., Schonbrun E. & Park W. Tunable superlensing by a mechanically controlled photonic crystal. J. Opt. Soc. Am. B: Opt. Phys. 23, 479–484 (2006).

[b8] Yim S. & Sitti M. Shape-programmable soft capsule robots for semi-implantable drug delivery. IEEE Trans. Robot. 28, 1198–1202 (2012).

[b9] Tokudome Y., Suzuki K., Kitanaga T. & Takahashi M. Hierarchical nested wrinkles on silica-polymer hybrid films: stimuli-responsive micro periodic surface architectures. Sci. Rep. 2, 683 (2012). 23002424 10.1038/srep00683 PMC3448069

[b10] Bertoldi K., Reis P. M., Willshaw S. & Mullin T. Negative poisson’s ratio behavior induced by an elastic instability. Adv. Mater. 22, 361–366 (2010). 20217719 10.1002/adma.200901956

[b11] Babaee S. . 3D soft metamaterials with negative Poisson’s ratio. Adv. Mater. 25, 5044–5049 (2013). 23878067 10.1002/adma.201301986

[b12] Chen S. L. . Polymeric nanosprings by bicomponent electrospinning. Macromol. Mater. Eng. 294, 265–271 (2009).

[b13] Chen X. & Yin J. Buckling patterns of thin films on curved compliant substrates with applications to morphogenesis and three-dimensional micro-fabrication. Soft Matter 6, 5667–5680 (2010).

[b14] Couturier E., Dumais J., Cerda E. & Katifori E. Folding of an opened spherical shell. Soft Matter 9, 8359–8367 (2013).

[b15] Bendsoe M. P. & Sigmund O. Ch. 2, Topology Optimization: Theory, Methods and Applications (ed.) 86–90 (Springer, Berlin, 2004).

[b16] Zhou S. W. & Li Q. A variational level set method for the topology optimization of steady-state Navier-Stokes flow. J. Comput. Phys. 227, 10178–10195 (2008).

[b17] Lewinski T. & Sokolowski J. Optimal shells formed on a sphere. The topological derivative method. Report No. RR-3495, 62 (1998).

[b18] Plotnikov P. & Sokolowski J. Compressible Navier-Stokes equations: theory and shape optimization, 63–97 (Birkhäuser/Springer Basel AG, Basel, 2012).

[b19] Novotny A. A. & Sokołowski J. Topological derivatives in shape optimization: Interaction of Mechanics and Mathematics, 47–89 (Springer, Heidelberg, 2013).

[b20] Cadman J., Zhou S., Chen Y. & Li Q. On design of multi-functional microstructural materials. J. Mater. Sci. 48, 51–66 (2013).

[b21] Verheyen H. F. The complete set of Jitterbug transformers and the analysis of their motion. Comput. Math. Appl. 17, 203–250 (1989).

[b22] Nguyen-Thoi T., Liu G., Lam K. & Zhang G. A face‐based smoothed finite element method (FS‐FEM) for 3D linear and geometrically non‐linear solid mechanics problems using 4‐node tetrahedral elements. Int. J. Numer. Meth. Eng. 78, 324–353 (2009).

[b23] Nasto A. & Reis P. M. Localized Structures in Indented Shells: A Numerical Investigation. J. Appl. Mech. 81, 121008 (2014).

[b24] Hutchinson J. On the postbuckling behavior of imperfection-sensitive structures in the plastic range. J. Appl. Mech. 39, 155–162 (1972).

[b25] Chryssanthopoulos M., Baker M. & Dowling P. Imperfection modeling for buckling analysis of stiffened cylinders. J. Struct. Eng. 117, 1998–2017 (1991).

[b26] Sadovský Z., Teixeira A. & Guedes Soares C. Degradation of the compressive strength of rectangular plates due to initial deflection. Thin Wall. Struct. 43, 65–82 (2005).

[b27] Ho D. Buckling load of non-linear systems with multiple eigenvalues. Int. J. Solids. Struct. 10, 1315–1330 (1974).

[b28] Sundar S. & Bhagavan B. K. Generalized eigenvalue problems: Lanczos algorithm with a recursive partitioning method. Comput. Math. Appl. 39, 211–224 (2000).

[b29] Schenk C. A. & Schueller G. I. Buckling analysis of cylindrical shells with random geometric imperfections. Int. J. Nonlinear Mech. 38, 1119–1132 (2003).

[b30] Hanssen A., Hopperstad O., Langseth M. & Ilstad H. Validation of constitutive models applicable to aluminium foams. Int. J. Mech. Sci. 44, 359–406 (2002).

[b31] Ding K. & Ye L. Simulation of multiple laser shock peening of a 35CD4 steel alloy. J. Mater. Process. Technol. 178, 162–169 (2006).

[b32] Gielis J. A generic geometric transformation that unifies a wide range of natural and abstract shapes. Am. J. Bot. 90, 333–338 (2003). 21659124 10.3732/ajb.90.3.333

[b33] Kiper G. Ch. 16, New Trends in Mechanism Science Pisla Doina, Ceccarelli Marco, Husty Manfred & Corves Burkhard . (ed.) 137–145 (Springer: Netherlands, , 2010).

[b34] Wan F. Y. M. & Weinitschke H. J. On shells of revolution with the Love-Kirchhoff hypotheses. J. Engrg. Math. 22, 285–334 (1988).

[b35] Paulose J. & Nelson D. R. Buckling pathways in spherical shells with soft spots. Soft Matter 9, 8227–8245 (2013).

[b36] King P. H. . Towards molecular computing: Co-development of microfluidic devices and chemical reaction media. Biosystems 109, 18–23 (2012). 22306034 10.1016/j.biosystems.2012.01.003

