# Supplementary material for: Buckling-induced retraction of spherical shells: A study on the shape of aperture
Source: Sci Rep. 2015 Jun 22;5:11309. doi: 10.1038/srep11309 (PMC4476044; doi:10.1038/srep11309)
Supplement: Supplementary Information [file srep11309-s1.doc]

Buckling-induced retraction of spherical shells: A study on the shape of aperture

Sen Lin, Yi Min Xie, Qing Li, Xiaodong Huang, Shiwei Zhou

Supplementary information

In Fig. S1, the FEA snapshots of the strain energy distribution during buckling of the shell model for LE and NH material models were presented. At the same stage of buckling, the distributions of strain energy in the LE and NH models were qualitatively similar. To further compare the simulations with these two methods quantitatively, we used the total strain energy as the measurement (Fig. S2). The quantitative results were nearly identical. Therefore, the formula from linear theory could be used here. Note the Young's modulus *E*=695,400 Pa and a Poisson's ratio *ν*=0.48 were used for LE model.

| Buckliball | | A | B | C | D |
| --- | --- | --- | --- | --- | --- |
| LE | 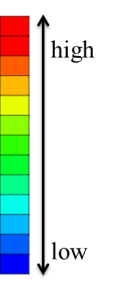 | 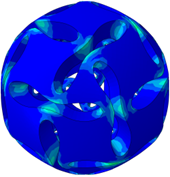 | 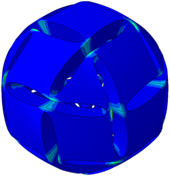 | 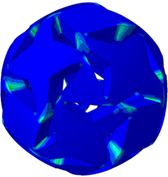 | 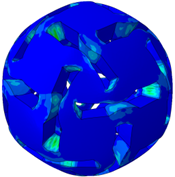 |
| Neo-Hookean |  | 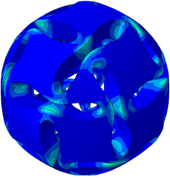 | 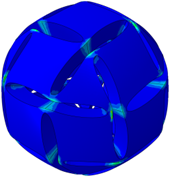 | 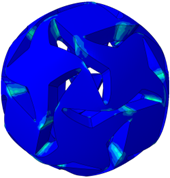 | 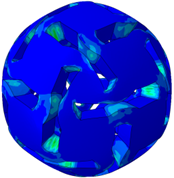 |
| **Fig.S1.** The distribution of strain energy on the surface of buckled shells by using LE and NH material model. | | | | | |

|  |
| --- |
| **Fig.S2** Quantitative comparison of strain energy versus volume retraction ratio between LE and NH material models. |

For the material test, we firstly designed and prepared the model for material testing. The size of specimen structure in the shape of a typical dog-bone shape (Figs. S3a-b) was adjusted accordingly to fit the testing equipment (Fig. S3c). Similar to Buckliball-B, the specimens were prepared by using 3D printing technique. From the laboratory tests, the average density of these samples was found to be 1,133.48 kg/m^3^ and the Poisson’s ratio was 0.48. To ensure no crack appears at the shoulders fixed by the grips when the samples were stretched, they were protected by two aluminum splints. The tests were conducted by moving upward the top grip at a ﬁxed rate of 10 mm per minute, equivalent to a strain rate 3.335x10^-3^ per second. The specimen was marked with white markers distinguishing from the specimen’s black color. Therefore a video extensometer was capable of capturing images of the deforming sample continuously. The stress-strain curve can be plotted until the specimen was fractured. The tests were repeated 3 times and the Young’s modulus was found to be 695,400 Pa.

| 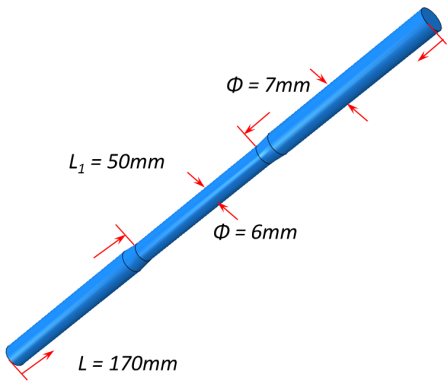 | 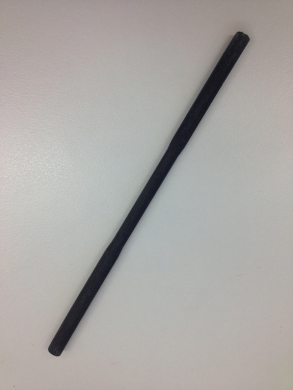 | 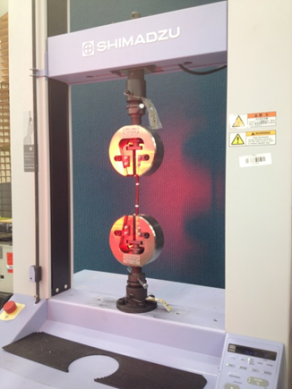 |
| --- | --- | --- |
| (a) | (b) | (c) |
| **Fig. S3** (a) The key sizes of the tested sample. (b) The sample made by 3D printing technique. (c) SHIMADZU Compression and Tension Equipment (10kN capacity) used in the test. Photo courtesy of Sen Lin. | | |
